# Supplementary material for: A Critical View of the Application of the APEX Software (Aqueous Photochemistry of Environmentally-Occurring Xenobiotics) to Predict Photoreaction Kinetics in Surface Freshwaters
Source: Molecules. 2019 Dec 18;25(1):9. doi: 10.3390/molecules25010009 (PMC7017383; doi:10.3390/molecules25010009)
Supplement: Supplementary file 1 [file molecules-25-00009-s001.zip › 3.2.4_gcc-4.4.0.zip!/3.2.4_gcc-4.4.0/3.2.4_gcc-4.4.0/doc/octave/html/interpreter/Organization-of-Functions.html]

Organization of Functions - Untitled


Previous: Commands,
Up: Functions and Scripts


---

### 11.11 Organization of Functions Distributed with Octave

Many of Octave's standard functions are distributed as function files.
They are loosely organized by topic, in subdirectories of
octave-home/lib/octave/version/m, to make it easier
to find them.

The following is a list of all the function file subdirectories, and the
types of functions you will find there.

audio: Functions for playing and recording sounds. control: Functions for design and simulation of automatic control systems. elfun: Elementary functions. finance: Functions for computing interest payments, investment values, and rates of return. general: Miscellaneous matrix manipulations, like `flipud`, `rot90`, and `triu`, as well as other basic functions, like `ismatrix`, `nargchk`, etc. image: Image processing tools. These functions require the X Window System. io: Input-output functions. linear-algebra: Functions for linear algebra. miscellaneous: Functions that don't really belong anywhere else. optimization: Minimization of functions. path: Functions to manage the directory path Octave uses to find functions. pkg: Install external packages of functions in Octave. plot: Functions for displaying and printing two- and three-dimensional graphs. polynomial: Functions for manipulating polynomials. set: Functions for creating and manipulating sets of unique values. signal: Functions for signal processing applications. sparse: Functions for handling sparse matrices. specfun: Special functions. special-matrix: Functions that create special matrix forms. startup: Octave's system-wide startup file. statistics: Statistical functions. strings: Miscellaneous string-handling functions. testfun: Perform unit tests on other functions. time: Functions related to time keeping.
